# Supplementary material for: Comparison and Analysis of the Genomes of Three Strains of Botrytis cinerea Isolated from Pomegranate
Source: Microorganisms. 2025 Jul 8;13(7):1605. doi: 10.3390/microorganisms13071605 (PMC12298828; doi:10.3390/microorganisms13071605)
Supplement: Supplementary file 1 [file microorganisms-13-01605-s001.zip › microorganisms-3670647-supplementary.pdf]

Table S1. Enzymes involved in pectin degradation identified in the genomes of three *B. cinerea* strains isolated from pomegranate fruits.

BcPgIs-1	BcPgIs-3	MIC
Polygalacturonase	Polygalacturonase	Polygalacturonase
Polygalacturonase	Polygalacturonase	Polygalacturonase
Polygalacturonase	Polygalacturonase	Polygalacturonase
Polygalacturonase	Polygalacturonase	Polygalacturonase
Polygalacturonase	Polygalacturonase	Polygalacturonase
Pectin lyase	Pectin lyase	Pectin lyase
Pectin lyase	Pectin lyase	Pectin lyase
Pectin lyase	Pectin lyase	Pectin lyase
Pectin lyase	Pectin lyase	Pectin lyase
Pectin lyase	Pectin lyase	Pectin lyase
Pectin lyase	Pectinesterase	Pectin lyase
Pectin lyase	Pectinesterase	Pectin lyase
Pectinesterase	Pectinesterase	Pectinesterase
Pectinesterase	Pectinesterase	Pectinesterase
Pectinesterase	Pectinesterase	Pectinesterase
Pectinesterase	Pectinesterase	Pectinesterase
Alpha-galacturonidase	Rhamnogalacturonase	Pectinesterase
Alpha-galacturonidase	Rhamnogalacturonase	Pectinesterase
Alpha-galacturonidase	Rhamnogalacturonase	Alpha-galacturonidase
Rhamnogalacturonase	Acyl-protein thioesterase	Alpha-galacturonidase
Rhamnogalacturonase	Alpha-L-rhamnosidase	Alpha-galacturonidase
Rhamnogalacturonase		Rhamnogalacturonase
Acyl-protein thioesterase		Rhamnogalacturonase
Acyl-protein thioesterase		Rhamnogalacturonase
Alpha-L-rhamnosidase		Acyl-protein thioesterase
		Acyl-protein thioesterase
		Alpha-L-rhamnosidase

Table S2. Enzymes involved in lignin degradation identified in the genomes of three *B. cinerea* strains isolated from pomegranate fruits.

BcPgIs-1	BcPgIs-3	MIC
Laccase	Laccase	Laccase
Laccase	Laccase	Laccase
Laccase	Laccase	Laccase
Laccase	Laccase	Laccase
Laccase	Laccase	Laccase
Laccase	Cellobiose dehydrogenase	Laccase
Laccase	Cellobiose dehydrogenase	Laccase
Laccase	Cellobiose dehydrogenase	Laccase
Laccase	Oxidoreductase	Laccase
Laccase	Diphenol oxidase	Laccase
Cellobiose dehydrogenase	Diphenol oxidase	Laccase
Cellobiose dehydrogenase	Vanillyl-alcohol oxidase	Cellobiose dehydrogenase
Cellobiose dehydrogenase	p-Benzoquinone reductase	Cellobiose dehydrogenase
Galactose oxidase		Cellobiose dehydrogenase
Galactose oxidase		Oxidase
Galactose oxidase		Oxidase
Oxidase		Oxidase
Oxidase		Oxidase
Vanillyl-alcohol oxidase		Oxidase
p-Benzoquinone reductase		Glucose-methanol-choline oxidoreductase
		Vanillyl-alcohol oxidase
		p-Benzoquinone reductase

Table S3. Enzymes involved in cellulose degradation identified in the genomes of three *B. cinerea* strains isolated from pomegranate fruits.

BcPgIs-1	BcPgIs-3	MIC
Cellulase	Endoglucanase	Endoglucanase
Cellulose	Endoglucanase	Endoglucanase
Endoglucanase	Endoglucanase	Endoglucanase
Endoglucanase	Endoglucanase	Endoglucanase
Endoglucanase	Glycoside hydrolase	Endoglucanase
Exoglucanase	Glycoside hydrolase	Endoglucanase
Glycoside hydrolase	Lytic polysaccharide monooxygenase	Glycoside hydrolase
Glycoside hydrolase		Glycoside hydrolase
Lytic polysaccharide monooxygenase		

Table S4. Enzymes involved in xylan degradation identified in the genomes of three *B. cinerea* strains isolated from pomegranate fruits.

BcPgIs-1	BcPgIs-3	MIC
Endo-1,4-beta-xylanase	Endo-1,4-beta-xylanase	Endo-1,4-beta-xylanase
Endo-1,4-beta-xylanase	Endo-1,4-beta-xylanase	Endo-1,4-beta-xylanase
Endo-1,4-beta-xylanase	Endo-1,4-beta-xylanase	Endo-1,4-beta-xylanase
Endo-1,4-beta-xylanase	Endo-1,4-beta-xylanase	Endo-1,4-beta-xylanase
Alkaline xylanase	Alkaline xylanase	Alkaline xylanase
Alpha-N-arabinofuranosidase	Alpha-N-arabinofuranosidase	Alpha-N-arabinofuranosidase
Alpha-N-arabinofuranosidase	Alpha-N-arabinofuranosidase	Alpha-N-arabinofuranosidase
Acetylxylan esterase	Acetylxylan esterase	Acetylxylan esterase
Acetylxylan esterase	Acetylxylan esterase	Acetylxylan esterase
1,3-beta-galactosidase	1,3-beta-galactosidase	1,3-beta-galactosidase
1,4-beta-xylosidase	1,4-beta-xylosidase	1,4-beta-xylosidase
1,4-beta-xylosidase	1,4-beta-xylosidase	1,4-beta-xylosidase
Glycoside hydrolase	1,4-beta-xylosidase	1,4-beta-xylosidase
Xylan alpha-1-2-glucuronosidase		Glycoside hydrolase
		Xylan alpha-1-2-glucuronosidase

Table S5. Phenotype matches obtained from blastp analysis against the pathogen-host interaction database (PHI-base) for genes found in the genomes of three *B. cinerea* strains isolated from pomegranate fruits.

B. cinerea strain	Sequence	Phenotype
BcPgIs-1	FUN_003004-T1	Reduced virulence
	FUN_000057-T1	Reduced virulence
	FUN_011469-T1	Reduced virulence
	FUN_000702-T1	Loss of pathogenicity
	FUN_001816-T1	Loss of pathogenicity, reduced virulence
	FUN_006454-T1	Reduced virulence
	FUN_009511-T1	Reduced virulence
	FUN_003110-T1	Loss of pathogenicity
BcPgIs-3	FUN_007008-T1	Reduced virulence
	FUN_002146-T1	Reduced virulence
	FUN_010843-T1	Reduced virulence
	FUN_004250-T1	Reduced virulence
	FUN_001878-T1	Reduced virulence
	FUN_006459-T1	Reduced virulence
	FUN_009579-T1	Reduced virulence
	FUN_003142-T1	Loss of pathogenicity
MIC	FUN_008163-T1	Reduced virulence
	FUN_000245-T1	Reduced virulence
	FUN_010711-T1	Reduced virulence
	FUN_002577-T1	Reduced virulence
	FUN_001831-T1	Loss of pathogenicity
	FUN_006454-T1	Reduced virulence
	FUN_009472-T1	Reduced virulence
	FUN_003113-T1	Reduced virulence
